# Neuroferritinopathy: From ferritin structure modification to pathogenetic mechanism

**DOI:** 10.1016/j.nbd.2015.02.007

**Published:** 2015-09

**Authors:** Sonia Levi, Ermanna Rovida

**Affiliations:** a University Vita-Salute San Raffaele, Division of Neuroscience, 20132 Milano, Italy; b San Raffaele Scientific Institute, Division of Neuroscience, 20132 Milano, Italy; c Institute for Genetic and Biomedical Research, National Research Council, Via Fantoli 16/15, 20138 Milan, Italy

**Keywords:** NF, Neuroferritinopathy, *FTL1*, Ferritin light chain gene, FtL, Ferritin light chain protein, FtH, Ferritin heavy chain protein, Tg, transgenic, WT, wild type, ROS, reactive oxygen species, MRI, magnetic resonance imaging, Neuroferritinopathy, Neurodegenerative disorder, Ferritin, Iron, Oxidative damage

## Abstract

Neuroferritinopathy is a rare, late-onset, dominantly inherited movement disorder caused by mutations in L-ferritin gene. It is characterized by iron and ferritin aggregate accumulation in brain, normal or low serum ferritin levels and high variable clinical feature. To date, nine causative mutations have been identified and eight of them are frameshift mutations determined by nucleotide(s) insertion in the exon 4 of L-ferritin gene altering the structural conformation of the C-terminus of the L-ferritin subunit. Acting in a dominant negative manner, mutations are responsible for an impairment of the iron storage efficiency of ferritin molecule. Here, we review the main characteristics of neuroferritinopathy and present a computational analysis of some representative recently defined mutations with the purpose to gain new information about the pathogenetic mechanism of the disorder. This is particularly important as neuroferritinopathy can be considered an interesting model to study the relationship between iron, oxidative stress and neurodegeneration.

## Neuroferritinopathy

The Neuroferritinopathy (NF) (OMIM, 606159, also labeled as hereditary ferritinopathies or NBIA3) (Curtis et al., 2001, Ohta and Takiyama, 2012) is a rare monogenic autosomal-dominant disease caused by mutations in the gene encoding the ferritin L-chain (*FTL1*) one of the two subunits of the main iron storage protein. The disease is classified as belonging to a growing collection of movement disorders named Neurodegeneration with Brain Iron Accumulation (NBIA). The NBIA are a group of degenerative extrapyramidal monogenic genetic diseases determining, in affected patients, radiological evidence of focal accumulation of iron in the brain, usually in the basal ganglia. They are characterized by early- or late onset with main symptoms that are problems encountered in the movement, spasticity and cognitive deficits (Levi and Finazzi, 2014). Among the causative genes, it can be distinguished between those encoding proteins directly involved in iron metabolism (*FTL1* and *CP*) and those encoding proteins responsible for other functions, such as: i) fatty acid metabolism and other mitochondrial functions (*PANK2*, *PLA2G6*, *C19orf12*, *COASY*, and *FA2H* genes); ii) lysosomal and autophagosome activity (*WDR45* and *ATP13A2* genes); and iii) a nuclear protein of still unknown function (*C2orf37* gene) (for an extensive review see Levi and Finazzi, 2014). A small subgroup of the identified NBIA cases is represented by NF, which is the only form inherited in an autosomal dominant manner.

From an epidemiological point of view, it is important to note that this disorder is extremely rare, thus so far, there is no available data on the prevalence of the disease in the population. However, due to overlapping of symptoms and MRI signs with the other forms of NBIA, it is conceivable that other incorrectly diagnosed cases may exist.

### Genetics

**Fig. 1:**
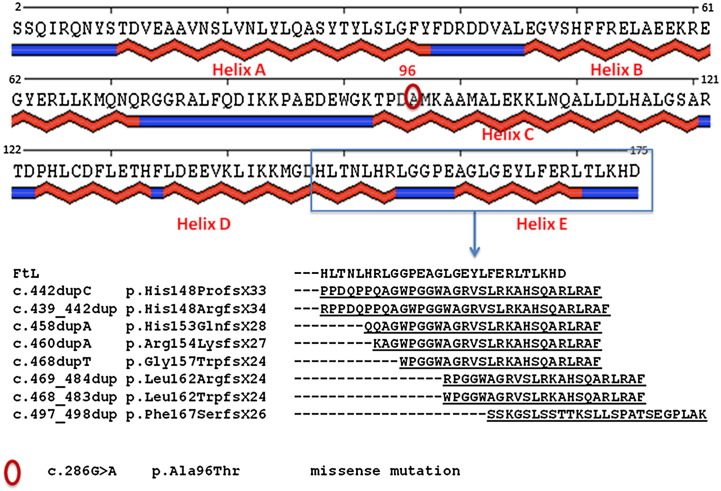
Schematic representation of L ferritin secondary structure and sequence alignment of FtL and NF variants. Human L ferritin sequence is matched with a schematic diagram of protein secondary structure elements that include five α-helices segments (red in upper figure), conventionally named with letters from A to E, connected by four loop elements and of two additional loops at the N and C termini (shown in blue). The red circle marks the position of residue 96, mutated in the unique missense variant (p.Ala96Thr). The blue rectangle highlight the sequence interval, from residue 148 to 175, involved in the so far detected frameshift variants. Frameshift mutations observed in patients are responsible for aminoacidic changes at the C-terminus starting from different sequence position depending on the duplication starting point. In panel B the alignment of the frameshift altered regions compared with the wild type FtL sequence is shown. Dashes correspond to unchanged position while modified residues are underlined.

NF was initially identified by Curtis among the members of a large family from the Cumbrian region of North England (Curtis et al., 2001). Using a genome wide linkage analysis, the authors identified the insertion of an adenine in position c.460 of exon 4 of the *FTL1* gene as the causative mutation of a previously unacknowledged neurodegenerative disorder that they named neuroferritinopathy (Curtis et al., 2001). Initially, the disease appeared to be confined to the population of North England, where more than 40 cases with the same mutation were identified, suggesting that they originated from a common founder (Chinnery et al., 2007). Subsequently, other cases were described in different parts of the world. Up to now, other 8 types of mutations have been identified in different ethnic groups (Vidal et al., 2003); (Mancuso et al., 2005);(Maciel et al., 2005); (Ohta et al., 2008); (Kubota et al., 2009); (Devos et al., 2009, Moutton et al., 2014, Nishida et al., 2014, Storti et al., 2013); they are reported in Table 1, adopting the HGVS nomenclature (den Dunnen and Antonarakis, 2000 and www.hgvs.org/mutnomen). The *FTL1* gene is located on chromosome 19q13.33 and it is composed by 4 exons and 3 introns. All the NF causative mutations, except one, are located on the exon 4 of the gene in a short DNA fragment of 58 nucleotides in length. They are single or multiple (from 2 to 16) nucleotides resulting in an altered sequence and length of the C-terminal portion of the encoded protein (Fig. 1). Interestingly, the mutation c.469_484dup, involving the duplication of 16 nucleotides, has been identified in two patients belonging to different and geographically distant ethnic groups, i.e. Japan (Ohta et al., 2008) and Italy (Storti et al., 2013). Together, all these observations insinuate that exon 4 of the *FTL1* might be recognized as a mutation hotspot. It must be noted that another region of about 70 nts in length characterized by high mutation frequency was identified in *FTL1* (Ferrari et al., 2006); (Luscieti et al., 2013). The region is located at 5′ UTR of the L-ferritin mRNA, corresponding to the iron responsive element sequence (IRE). It folds in a stem loop structure, and is involved in the iron-mediated post-trascriptional regulation of ferritin expression (Hentze et al., 2010). Anomalies in IRE sequence cause a disease named hereditary hyperferritinemia cataract syndrome (HHCS, OMIM# 600886). It is a rare autosomal dominant disease characterized by increased serum ferritin levels and early onset of bilateral cataract (Girelli et al., 1995). Affected individuals show high serum ferritin levels, with normal serum iron and transferrin saturation, and without signs of systemic or brain iron overload (Cazzola, 2002). The lack of post-transcriptional control of ferritin expression produces L-ferritin (FtL) accumulation in lens, where it induces cataract formation by altering the delicate equilibrium between other water-soluble proteins, such as crystallins (Levi et al., 1998).

Altered ferritin levels in brain are also reported for other neurodegenerative disorders, such as Parkinson's (PD) and Alzheimer's disease (AD). There are some controversies in the literature regarding the change in the concentration of ferritin in PD brain compared to controls, while an increase in brain ferritin amount in AD is well accepted (for a in-depth discussion on this topic see (Finazzi and Arosio, 2014)). Nevertheless, abnormal ferritin levels in PD and AD are associated to a loss of ferritin ability to maintain iron homeostasis (Finazzi and Arosio, 2014);(Connor et al., 1995) rather than a sequence variation of ferritin genes as in NF.

### Clinical features

So far, the largest cohort of NF patients was analyzed by Chinnery and coworkers (Chinnery et al., 2007). The authors collected data on 41 patients, all of them carrying the c.460dupA mutation, and showing an average age of onset of 39.4 years. The main clinical manifestations were those that characterized extrapyramidal disorders: chorea (50%), followed by focal lower limb dystonia (42.5%) and parkinsonism (7, 5%). Patients often showed typical facial anomalies due to the activation of the frontal muscles, in particular oromandibular dyskinesia. In general, there was asymmetry throughout the disease course: the early signs could disturb the first one side of the body and then the other, or they were intermittent. Progression was slow and eventually leading to aphonia, dysphagia and severe motor disability with subcortical/frontal cognitive dysfunction as a late feature (Chinnery et al., 2007).

Comparison of clinical evidences in patients carrying other mutations showed that all of them gave rise to similar symptoms, such as oro-buccal dyskinesia, chorea and dystonia. However, subtle phenotypic variations between them in terms of age of onset, progression of the disease or the presence of cognitive disorders were observed (Table 1) (for a full description of all cases see Keogh et al., 2013). Indeed, a recent study reported a new case with atypical presentation (Nishida et al., 2014). The authors described a family affected by chronic headaches and lacking the classical clinical symptoms at the early stage of the disease; however they developed progressive orolingual and arm dystonia, dysarthria, cerebellar ataxia, pyramidal tract signs, and psychiatric symptoms in a later stage (Nishida et al., 2014). Thus, it is conceivable that the disease can manifest with a variety of symptoms that have not yet been fully described.

### Diagnosis

Routine blood tests are usually normal in patients, except for serum ferritin, which is often lower than normal (Table 1) and thus can be a warning sign when the presence of disease is suspected. Lehn et al. (2012) reviewed all the published NF cases and calculated that 64% of affected males and 84% of females exhibited levels of serum ferritin lower than a cutoff value set to 30 g/L. Analysis of cerebrospinal fluid (CSF) did not show appreciable differences (Chinnery et al., 2007); (Wills et al., 2002, Lehn et al., 2012). However, the case described by Nishida et al. (2014) showed a remarkably low level of CSF ferritin (< 1 ng/ml), compared to values of control subjects (6.68 +/− 0.93 ng/ml), and despite a ferritin level of 20 g/L in serum.

Standard histochemical analyses of muscle biopsies are usually normal; however Chinnery et al. (2007) found a significant percentage of cytochrome c oxidase-negative fibers in 2 out of 9 analyzed patients, in addition to isolated or combined defects of respiratory chain complexes in 5 out of 6 analyzed patients.

**Fig. 2:**
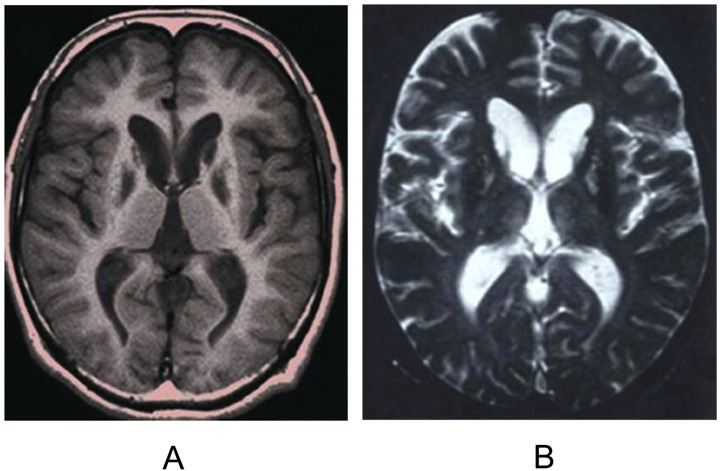
Brain MRI in Neuroferritinopathy. Brain MR images of a Japanese man carrying the *FTL1* c469_484dup mutation. Axial section at the level of the basal ganglia in the patient at 35 years of age. A A T1-weighted image (TR 400 msec/ TE 14 msec) shows symmetrical hypointense signals in the head of the caudate nucleus and globus pallidus. B A T2-weighted image (TR 800 msec/TE 30 msec) shows hypointense changes in the lenticular nucleus. Hyperintense signals can be observed in the putamen and the head of the caudate nucleus. Reproduced from Ohta E, Takiyama Y. MRI findings in neuroferritinopathy. Neurol Res Int. 2012;2012:197438.

Magnetic Resonance Imaging (MRI) is the most effective tool for NF diagnosis (see an example in Fig. 2); it is applicable during the whole course of the disease, with important detections also in the early stages. In fact, iron deposits in cerebellum, basal ganglia and motor cortex are detected by traditional “gradient echo sequences” (T2*), although more recently the “susceptibility weighted imaging” (SWI) is emerging as a viable alternative.

The first visible MRI change in early stage symptomatic, and also in some asymptomatic carriers, is a hypodensity in T2* and SWI at the level of the dentate nucleus, red nucleus, substantia nigra, putamen, globus pallidus, thalamus, caudate nucleus and the motor cortex (Chinnery et al., 2007); (Ohta and Takiyama, 2012). In this phase, the only visible abnormality in T2-weighted images is a slight lowering of the level signal of the nuclei of the base (Chinnery et al., 2007). With the progression of the disease, the T2-weighted images showed hyperintensity at the level of the basal ganglia due to edema and gliosis prior to degeneration (Chinnery et al., 2007); (Ohta and Takiyama, 2012); in later phases, hypointense areas are associated to the hyperintensity, due to the deposition of iron (Kruer and Boddaert, 2012). In more advanced stages, areas of cystic cavitation are formed, which may be preceded by hyperintensity on T1-weighted images, particularly in the putamen and globus pallidus; these may be associated with cerebellar atrophy and a slight cerebral atrophy (Kruer and Boddaert, 2012). However, since “the eye of tiger sign”, which is considered pathognomonic of PKAN disease, was detected in three NF patients (McNeill et al., 2008), it must be considered that MRI analysis cannot be exhaustive and that the diagnostic evidence should always come from genetic testing.

### Pathology

The typical neuropathological features of the disease are related to the presence of ferritin and iron aggregates in neural tissue. The first histopathology data were reported for patients carrying the c.460dupA mutation (Curtis et al., 2001). The post-mortem brain inspection revealed the presence of spherical granules in the globus pallidus, forebrain and cerebellum. These granules were positive for both iron and ferritin staining. They were mainly extracellular but co-localized also within neurons, oligodendrocytes and microglia. Subsequent brain histopathological analysis of patients carrying c.442dupC and c. 497_498dupTC mutations (Vidal et al., 2004); (Mancuso et al., 2005) confirmed that the presence of intracytoplasmic and intranuclear aggregates of ferritin in the glial cells and in some neuronal subtypes, deposits of iron, gliosis and neuronal death represented the prevalent neuropathological findings in NF. In general neuronal aggregates were observed in putamen, globus pallidus, thalamus, in cerebellar granule and Purkinje cells, while glial cell aggregates were mainly detected in the caudate, putamen and globus pallidus. In these patients, the composition of aggregates was determined by immunostaining with anti-wild type FtL, anti-variant FtL and anti FtH antibodies (Vidal et al., 2004), suggesting that the three ferritins species were assembled in the full ferritin 24-mer. They also contained both Fe^2 +^ and Fe^3 +^, as revealed by Turnbull and Perls' Prussian blue staining, respectively (Mancuso et al., 2005). The presence of aggregates was detected not only in CNS but also in other tissues such as skin, liver, kidney and muscle (Mancuso et al., 2005, Vidal et al., 2004).

## Ferritin

**Fig. 3:**
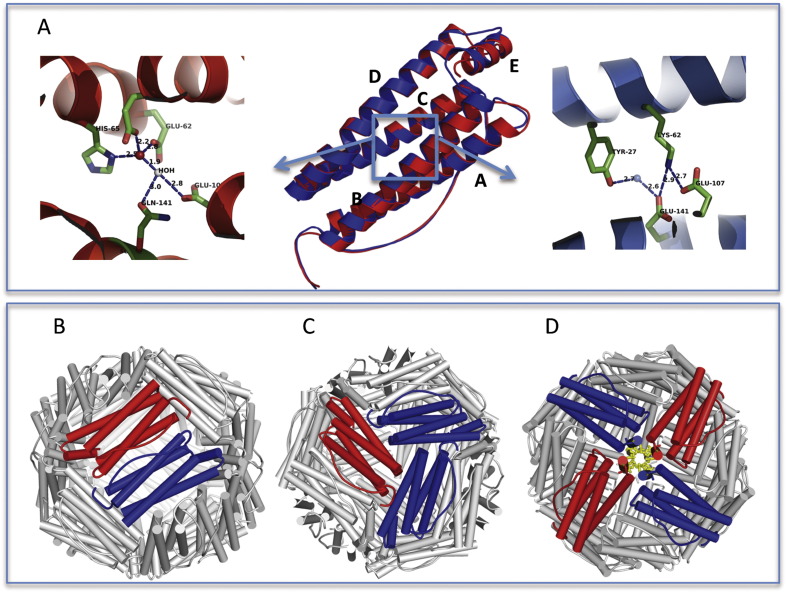
Three-dimensional structure of ferritin. Ferritin subunit is a four-helix bundle protein consisting of two couples of anti-parallel α-helices (A–B and C–D) connected by a long loop and a short C-terminal α-helix (E) important for 24-mer stabilization. Human L and H ferritin subunits share a 55% sequence identity and a remarkably similar 3D structure. A In central panel is shown a ribbon representation of superposed L (blue) and H (red) human ferritin subunits. Superposition of the two structures yielded a root mean square deviation (RMSD) of 0.5 Å, indicating an elevated level of structural similarity. The two subunits show a different residue arrangement in the center of four-helix bundle, corresponding to the blue box. Arrows departing from the box point to the expanded representation of the region in H-chain (panel left side) and L-chain (panel right side). In H chain, a ferroxidase center is formed by a set of iron coordinating residues, which are replaced by a salt bridge forming amino acids in the L chain. The functional protein is a 24-mer polymer made of variable proportion of H and L chains that assemble following a two-three- and four-fold symmetry. The assembled ferritin appears as a hollow spherical shell (panel B). Subunit interactions in the 24-mer generate 12 dimeric interfaces (a dimer formed by one H and one L chain is shown as an example in panel B), eight hydrophilic channels, each obtained from 3 subunits, at the three fold axes (panel C) and six hydrophobic channels, obtained from 4 subunits at the four-fold axes (panel D). Structures were obtained from Protein Data Bank (PDB code: 2FFX for L- chain, 1FHA for H-chain; Berman et al, 2000).

Ferritin is the ubiquitous protein of iron storage, evolutionary conserved from prokaryotes to mammals; it is characterized by a structure evocative of its function, consisting of a virtually spherical shell with an internal cavity that can accommodate up to 4500 iron atoms (Arosio and Levi, 2010). Its primary functional role is to sequester free iron from various cell compartments preventing its reaction with oxygen and the consequent formation of reactive oxygen species (ROS) whose oxidative harmful effect are well known (Graf et al., 1984). For this reason, ferritin is widely distributed in different tissues and cell compartments. Cytoplasmatic ferritin is most abundant in human cells (Arosio and Levi, 2010), however a mitochondrial form has also been identified and characterized (Levi et al., 2001). Cytosolic ferritin has a typical quaternary structure symmetrically assembled from of 24 subunits of two different types, termed H and L (Fig. 3B-C-D), which are present in different proportions depending on tissue type (Arosio and Levi, 2010). The H and L chains are encoded by human genes, located on chromosomes 11 and 19, respectively, and share about 55% amino acid sequence identity. The 3D structure of the two chains is very similar: a bundle of 4 helices, with a long loop that connects the helix B and helix C, and a fifth smaller helix called E at the C-terminus, which forms an angle of 60° respect to the bundle of 4 helices, and is directed towards the center of the cavity (Fig. 3A). The H chain has a ferroxidase center within the bundle, where the oxidation of iron occurs (Levi et al., 1988); (Lawson et al., 1991) (Fig. 3A, left panel). Most of residues in the H chain that create the binding site for the iron are different in the L chain and create a salt bridge that stabilizes the chain (Santambrogio et al., 1992) (Fig. 3A, panel right). The L chain facilitates the mineralization of the iron in the cavity supporting the ferroxidase activity of the H chain (Levi et al., 1994), in fact, both in vitro and in vivo studies demonstrated that heteropolymers of the two subunits incorporates iron more efficiently than the homopolymers (Levi et al., 1994). The iron enters in the cavity through eight channels formed by the hydrophilic helices C and D of 3 different subunits, along the 3-fold symmetry axis (Fig. 3C), which have a binding site for metals; through these channels, individual iron atoms can diffuse in and out of ferritin (Watt et al., 2010). In addition to the hydrophilic channel there is another type of channel, along the 4-fold axis, formed by helices E of 4 different subunits, with hydrophobic properties because rich in leucine residues (Fig. 3D). The calculation of the local electrostatic potential suggests that this channel may be involved in the outflow of protons that occurs following the incorporation of iron into the cavity (Douglas and Ripoll, 1998).

### Neuroferritinopathy variants

Among the nine FtL variants identified so far, there is only a single case of a missense mutation (p.Ala69Thr), while all the others are responsible for a frameshift at C-terminus of the L chain resulting in a partial or complete sequence change of the E-helix (Table 1 and Fig. 1). Variants can be roughly divided into two groups: 1) the frameshift is upstream of the loop connecting D-helix to E-helix, thus the aminoacidic changes involve a long sequence stretch (26 to 33 residues) but the total protein length is similar to wild type (the first 5 variants in Fig. 1) and 2) the frameshift is downstream of the loop (variants 6 to 8 in Fig. 1) and the sequence length is from 8 to16 residues longer than wild type.

**Fig. 4:**
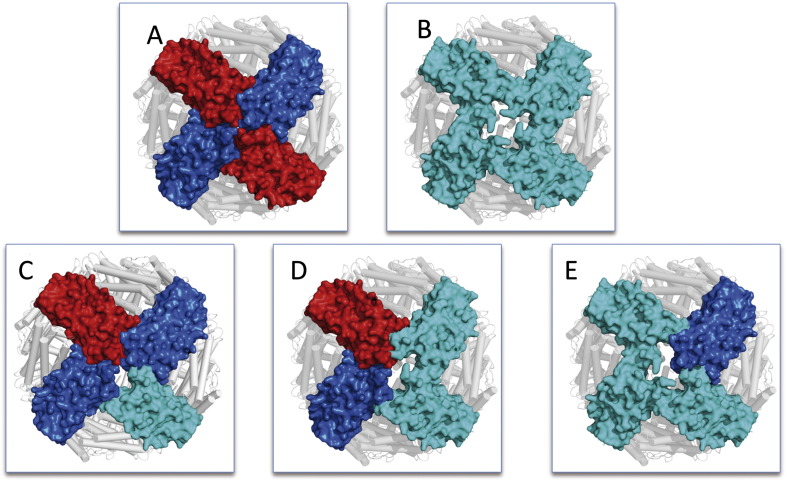
Molecular surface representation of ferritin subunits forming the 4-fold axis hydrophobic pore. A Representative wild type pore formed by two FtL (blue) and two FtH (red) chains. The wild type channel appears very narrow due to the presence of tightly packed hydrophobic residues. B Putative pore formed by 4 identical FtLPhe167SerfsX26 subunits (cyan). The opening of the pore is largely increased, altering the iron permeability of the ferritin shell. In patients carrying FtL mutation, two different L chain alleles (wild type and mutated), in addition to the wild type H chain, are expressed; therefore, mixed heteropolymers are likely to occur. Three putative combinations of FtL (blue), FtH (red) and FtLPhe167SerfsX26 (cyan) subunits, have been reconstructed in panels C, D, and E, increasing the proportion of mutated chain. The pore width increases proportionally to the number of variant chains participating in the tetramer assembly. (The structure of FtLPhe167SerfsX26 variant was obtained from Protein Data Bank. PDB code: 2KXU).

Between them, the variant p.Phe167SerfsX26 is best characterized at a biochemical and structural level (Baraibar et al., 2008);(Luscieti et al., 2010). X-ray crystal structure of the mutated monomer revealed the ferritin typical four helix bundle fold but missing the C-terminal E-helix that could not be solved due to the disorder and/or instability of the mutated peptide replacing the C-terminal helix (Luscieti et al., 2010). This has a deleterious effect on the 4 fold channel geometry and permeability, leading to mishandling of iron in ferritin. The structural effect of this mutation is depicted in Fig. 4, where the molecular surface of different types of tetramers within the ferritin 24-mer is shown. The wild type 4 fold channel (Fig. 4A) formed by four wild type subunits (FtH chains in red and FtL chains in blue) appears tightly packed and closed due to the presence of an arrangement of hydrophobic residues. A fully mutated tetramer instead (Fig. 4B) opens up a large pore at the four-fold channel, about 14 Å wide, dramatically altering the iron permeability of the ferritin shell. In these patients two different L chain alleles (wild type and mutated), in addition to the wild type H chain, are expressed; therefore, mixed heteropolymers including the three subunit types, are likely to be more representative of the in vivo condition. Within each 24-mer, the four-fold axis pore can be formed by different combinations of the variant FtL with the wild type L and H subunits (Fig. 4C-D-E). As depicted in Fig. 4, the pore width increases proportionally to the number of variant FtL chains participating in the tetramer assembly (cyan in Fig. 4). It is therefore apparent that even a small amount of mutant incorporation will affect iron management ability of the protein.

It is not known if the similar phenotypic effects of the other FtL frameshift variants (Table 1) can be attributed to a similar structural and functional impairment. Biochemical characterization of these variants is made difficult by the instability of the expressed proteins (Levi, unpublished data). To gain insight into the structural and functional effects of aforesaid mutations, we applied a predictive computational analysis to three representative variants p.Arg154LysfsX27, p.His148ProfsX33, p.Leu162ArgfsX24.

**Fig. 5:**
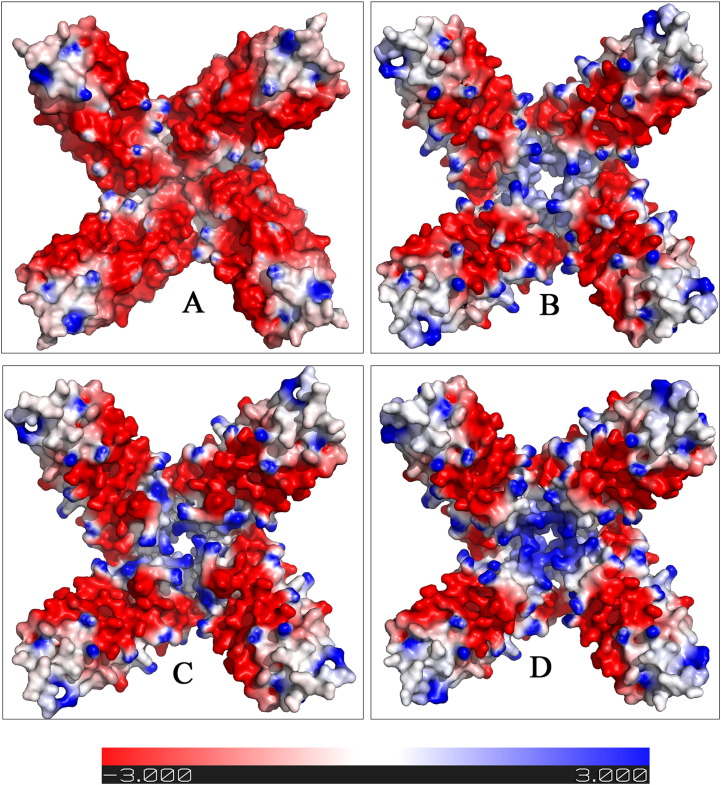
Electrostatic potential distribution of the inner surface of homopolymeric 4 fold axis tetramers. The tetrameric assembly of four identical subunits was reconstructed starting from the wild type FtL subunit (panel A) and the variants p.Arg154LysfsX27, His148ProfsX33 and His148ProfsX33 (panels B, C, D, respectively). In wild type FtL tetramer (panel A), a prevalence of negative charges (red surface) is noticeable and is required for the iron nucleation inside the cavity. All the analyzed variants show an increased distribution of neutral or positive charges (white to blue surface in panels B-C-D) around the four-fold channel, and this can be responsible for a repulsive effect on iron complex. In addition, the reconstruction of the four-fold tetramers showed, for all variants, a wider pore compared to wild type, likely to alter the ferritin shell permeability. The potential scale ranges from − 3kT/e to + 3kT/e from red to blue. The PyMol molecular visualization system (The PyMOL Molecular Graphics System, Schrödinger, LLC) was used for structure visualization, analysis, comparison and for preparation of images.

Molecular models were obtained by threading approach (Roy et al., 2010). In all cases, the mutated C-terminal peptides were predicted to assume a helical conformation superposed to the wild type E helix and further protruding into the cavity according to the extension of the C-terminal sequence. Electrostatic potential calculations (Honig and Nicholls, 1995) were performed for the obtained models and then represented as surface distribution for reconstructed homo-tetramers of each variants and for FtL. In Fig. 5 the distribution of the potential on the inner surface of the assembled protein for the FtL (Fig. 5A) and for the variants is shown (Fig. 5B-C-D). The prevalence of negative charges on the FtL (red surface in Fig. 5A) is a requirement for the iron nucleation inside the cavity. All the analyzed variants show a cluster of positive charges (blue surface in Fig. 5B-C-D) around the four-fold channel, that can be responsible for a repulsive effect on iron complex. In addition, the reconstruction of the four-fold tetramer showed, for all variants, a loose channel likely to alter the ferritin shell permeability. Although the modeling of the variant subunits might not reflect the actual state of the protein under physiological conditions, it provides a key for interpretation of the mutant effect. The change at the subunit C-terminus implies a modification of the microenvironment of the ferritin cavity that loses its ability to correctly process the iron deposition and retention. As for the unique missense variant related to NF onset (p.Ala96Thr) (Maciel et al., 2005), its effect can be hardly explained on the basis of the structural or functional impairment. In fact the amino acidic substitution is predicted as tolerated by most reliable methods of protein mutant prediction such as SIFT (Ng and Henikoff, 2003) and PolyPhen2 (Adzhubei et al., 2013).

## Advancement on pathogenetic mechanism studies

Several in vitro and in vivo studies were performed in an attempt to understand the pathogenetic mechanism of the disease. Most of them were carried out on the p.Phe167SerfsX26 variant (referred as FtL_Phe167SerfsX26_ in this section) (Fig. 1) as the in vitro stability of the protein product is higher than for other variants and adequate for performing biochemical analyses.

### In vitro studies

Biochemical study on the aggregates purified from patient tissues identified the presence of all the three subunit types: FtH, FtL and FtL _Phe167SerfsX26_ peptides, indicating that the variant peptide is able to assemble in vivo into the 24-mer molecule (Vidal et al., 2004). Indeed, the biochemical and crystallographic characterization of the recombinant homopolimer FtL_Phe167SerfsX26_ showed that it exists as a 24-mer molecule with an altered conformation of the C-terminus and a large disruption of the normally tiny four-fold axis pores (Luscieti et al., 2010); (Baraibar et al., 2010) (Fig. 4). To reproduce the typical form of ferritin, the authors analyzed in vitro reconstructed ferritin heteropolymers composed of 20 to 23 H-chains and 4 to 1 either wild type or variant L-chains (Luscieti et al., 2010). All heteropolymers exhibited a strongly reduced capacity to incorporate iron and a reduced physical stability. Thus, it was demonstrated that the presence of few mutated L-chains was sufficient to alter the permeability of 1–2 of the 6 hydrophobic channels and modify ferritin capacity to incorporate iron. These data highlight the dominant-negative action of the mutations, which explains the dominant transmission of the disorder (Luscieti et al., 2010).

Other biochemical studies on FtL_Phe167SerfsX26_ functionality were based on the in vitro assembly of wt and variant L chains in homopolymeric form (Baraibar et al., 2010). TEM analysis showed that both homopolymers have spherical structure and dimensions similar to those of human ferritin (outer diameter approximately 110 Å). Iron incorporation experiments for the two homopolymers showed a similar functionality at low iron concentrations (about 1500 iron atoms per 24-mer). When iron proportion was increased up to 4000 atoms per homopolymer, only the FtL incorporated iron and remained soluble, while the FtL_Phe167SerfsX26_ quickly precipitated (Baraibar et al., 2008); (Baraibar et al., 2010). These results were then confirmed by the same authors for heteropolymers containing the mutated peptide, which showed a greater propensity to iron-induced precipitation and reduced functionality (Muhoberac et al., 2011) compared to FtL. Further studies also reported prominent in vitro and in vivo propensity of the FtL_Phe167SerfsX26_ to be oxidized and pointed out the key role of oxidative stress for NF pathogenesis (Baraibar et al., 2012).

### Cellular models

Cellular models of c.460dupA and c.497_498dupTC were developed in HeLa and neuroblastoma SH-SY5Y cells by expressing the mutated L chains under the control of tetracycline (Cozzi et al., 2006); (Cozzi et al., 2010). Both type of mutations led to same results, suggesting that similar pathogenetic mechanisms are engaged by different structural alterations of ferritin. Both variants were expressed and assembled in the heteropolymer although at low proportion (an average of less than four subunits for polymer); their expression was associated with an increase of endogenous ferritin chains and a decrease of Transferrin Receptor1 (TfR1) expression. As these parameters are modulated by iron availability in cytosolic compartment, the obtained results clearly indicated the inefficiency of ferritin variants to incorporate iron. The incorporation of variant peptides in the assembled 24-mer resulted in a reduced ferritin functionality, thus confirming the dominant negative effect of the mutations (Cozzi et al., 2010). In addition, the containing-altered-peptide ferritins were degraded much faster than the FtL, primarily via proteasome, further increasing the iron release in cytoplasm (Cozzi et al., 2006); (Cozzi et al., 2010). Indeed, an increase of the redox-active labile iron pool (LIP) was detected after iron treatment, with consequent enhancement of ROS production after treatment with H_2_O_2_, as well as a defect in the proteasome activity.

Both cell lines developed aggregates, positive to L-ferritin and iron staining, which grew in number and size after iron addition (Cozzi et al., 2010). More importantly, these aggregates were not strictly associated to cell death: they became evident after 10 days of variant expression and were not associated to cells that were undergoing apoptosis. The addition of iron chelators or antioxidants restored proteasome activity and reduced aggregate formation (Cozzi et al., 2010). Overall data strongly suggested that cellular iron imbalance and oxidative damage were the primary causes of cell mortality, while protein aggregate formation was a secondary effect.

### Fibroblasts from a patient carrying the c.497_498dupTC mutation

Berbeito et al. had the opportunity to analyze skin-derived primary cells from a NF patient (Barbeito et al., 2010). These cells showed an altered management of iron and ferritin and an accumulation of oxidative stress markers as it was observed both in patients and in transgenic mice (Vidal et al., 2008). Under basal conditions, patient's fibroblasts showed a significant increase in the amount of total iron and in the expression of H-, L- and L-variant ferritin chains compared to control fibroblasts. TfR1 expression and the IRE-binding capacity of IRP decreased consistently with an increase of intracellular free iron, as previously reported for cellular models. However, significant differences in LIP level were not reported. This apparent discrepancy could be explained by the fact that even small LIP variations, not precisely detectable by the common assays, are sufficient to stimulate the iron-mediated control of protein expression (i.e. TfR1 and ferritins). In addition, the level of ROS was significantly enhanced in NF compared to controls fibroblasts (Barbeito et al., 2010).

### Animal models

A transgenic mouse was obtained by expressing the human cDNA of *FTL1* with the mutation c.497_498dupTC. The expression of the transgene in the animal caused the formation of nuclear and cytoplasmic ferritin aggregates in CNS and other organs (Vidal et al., 2008). Similarly to patients, the size and the number of nuclear aggregates increased with the aging of the animal (Vidal et al., 2004). The mouse model showed a progressive neurological phenotype, decreased mobility and a reduced life expectancy. In addition, it showed increased amount of iron in the brain and altered levels of associated proteins: the H and L ferritins expression was amplified while TfR1 level decreased. The transgenic mouse also showed accumulation of oxidized mitochondrial DNA but no significant damage to the nuclear DNA in brain (Deng et al., 2010) even if markers of oxidative stress such as lipid peroxidation and protein carbonylation were detected (Barbeito et al., 2009).

Further insight into the NF pathogenetic mechanism was recently obtained from the characterization of new transgenic mouse models for the same *FTL1* mutation. In this work mice models were obtained utilizing a PGK promoter based expression for generation of transgenic mice in FVB and C57BL/6 J strains (Maccarinelli et al., 2014). FVB transgenic mice (Tg) showed high accumulation of the FtL_Phe167SerfsX26_ in brain associated with increased iron deposition during age and with signs of oxidative damage. Interestingly, the C57BL/6 Tg mice preserved an evident oxidative alteration in the brain detectable at 12 months of age, although they showed a reduced FtL_Phe167SerfsX26_ expression and iron accumulation compared to the FVB-Tg mice. Ultrastructural analyses of brain tissues revealed an accumulation of lipofuscin granules associated with iron deposits, particularly enriched in the cerebellum and striatum of the C57BL/6 transgenic mice, and localized in cytoplasm. In contrast with the mouse model described by Vidal et al. (2008), no evidence of the presence of aggregates in the nucleus and in extracellular compartment was reported (Maccarinelli et al., 2014). In addition, post-natal hippocampal neurons obtained from C57BL/6 Tg mice showed higher vulnerability to chronic iron overload and/or acute oxidative stress then wild-type neurons, indicating a major propensity to cell death. Furthermore, behavioral studies showed a progressive impairment in motor coordination of Tg mice (Maccarinelli et al., 2014).

### Proposed pathogenetic mechanisms

The main role of ferritin is exerted by its ability to sequester iron and keep it in a safe form inside its internal cavity, even though other functions have been recently reported that are not associated with its metal incorporation activity (reviewed in Finazzi and Arosio, 2014). Indeed, in the case of NF, overall in vitro and in vivo data, agree in suggesting a pathogenic mechanism based on the non-controlled augment of intracytosolic free iron, due to the missing crucial role of the ferritin in maintaining iron balance inside the cell. In fact, as confirmed by the molecular modeling data, all the NF causative mutations lead to the same final effect: the inefficiency of iron nucleation inside ferritin cavity. This functional impairment can derive both from an altered arrangement of the residues lining the four-fold hydrophobic channel and from a modification of the chemico-physical properties of the ferritin interior which perturb the optimal microenvironment for ferrihydrite deposit formation (Behera and Theil, 2014).

Whatever the cause, the unsafely stored iron triggers a cascade of event leading to a vicious cycle in which cytosolic free iron continuously induces the iron-dependent ferritin mRNA translation, producing three different subunits (FtH, FtL and variant FtL) that assemble to form ferritin molecules with a reduced capacity to store iron. In the meantime the free iron stimulates the formation of ROS, which promote oxidative damage of cellular components (Cozzi et al., 2010). The overloaded activity of proteasome system to degrade high levels of oxidized proteins leads to proteasome dysfunction and consequently to ferritin aggregates formation (Cozzi et al., 2010). In this work, it was also shown that the proteasome impairment in NF cellular model was a consequence of the oxidative damage and not a direct effect of free iron. In fact, the authors detected the rescue of the proteasome activity in the presence of an anti-oxidant agent and not of an iron chelator (Cozzi et al., 2010). Data obtained on Tg mice indicated that these aggregates form lipofuscin that accumulates in lysosomes where the degradation of ferritin occurs, leading to further release of redox-active free iron (see Maccarinelli et al., 2014). Interestingly, it has been described that lipofuscin itself may adsorb ferrous iron, thus constituting a redox-active surface that may catalyze the generation of hydroxyl radicals and amplification of the oxidative damage (Hohn and Grune, 2013). In the long run, lysosomes, congested by lipofuscin, may go through rupture with consequent release of hydrolytic enzymes and lipofuscin into cytosol (see Maccarinelli et al., 2014). Thus, both proteasome and lysosomal cellular degradative systems result impaired with the formation of aggregosomes in a self-maintained circle of harmful events.

The other pathogenetic model proposed by (Muhoberac and Vidal, 2013) contemplated the gain of toxicity function of mutated ferritins that easily precipitated, being surrounded by free iron. Indeed, Fe^2 +^ has a strong propensity to oxidation and precipitation at physiologic pH. This may cause the non-specific precipitation of ferritin and other proteins that further contribute to the formation of aggregates (Muhoberac and Vidal, 2013).

However, it should be taken into account that the iron/ferritin aggregates can be present in brain patients several years before the developing of symptoms, as revealed by MRI analysis (Keogh et al., 2012). In addition, they are present in extraneuronal tissues that do not always show dysfunction (Curtis et al., 2001);(Mancuso et al., 2005). Thus, the aggregates may be present in cells without giving rise to an evident phenotype and neuronal cells appear to be the most sensible cells to the pathogenetic mechanism. This suggests that, even if the aggregates formation is a component of pathogenetic mechanism, the main player in the NF pathogenesis is the alteration of oxidative status, of which the brain tissue is particularly sensible due to paucity of antioxidant-defenses of the neuronal cells. Thus, the therapeutic intervention should be aimed not only at chelating free iron but also at preventing the oxidative damage. Some attempts to induce iron depletion have been undertaken on a few patients by venesection; the treatment produced profound iron depletion without yielding a clinical benefit (Chinnery et al., 2007); (Kubota et al., 2009).

## Conclusions

The overall data indicate that the different NF causative mutations exert a similar outcome, impairing ferritin iron storage capacity. The pathologic effect is compatible with the cell life, however, in long term, the iron-dependent oxidative damage, particularly evident in brain tissue, induces harmful effects. Thus, NF represents an exceptional model to study the relationship between iron excess, oxidative stress and neurodegeneration, a paradigm that exists in many neurodegenerative disorders. The full understanding of NF etiopathogenesis in human is essential to define a rational approach that should allow, in the near future, to design a trial for a disease modifying treatment. Moreover, this knowledge can highlight common mechanisms in different pathological neurodegenerative processes.

In addition, considering that the rate of lipofuscin accumulation is reported to correlate negatively with longevity, elucidation of NF pathogenetic mechanism can also help to explain mechanisms occurring during the physiological process of aging.

## Figures and Tables

**Table 1:** List of reported cases and related mutations of neuroferritinopathy.

Families from:	DNA^a^ mutations	Protein variant^b^	Symptomatology	Serum ferritin^c^	References
Cumbrian region (UK)	c.460dupA	p.Arg154LysfsX27	Extrapyramidal dysfunction choreathetosis, dystonia, spasticity, rigidity	4–16 (N.R.)	Curtis et al. (2001)
Northwest of UK	c.460dupA	p.Arg154LysfsX27	Extrapyramidal dysfunction including palatal tremor and cognitive decline	60 (25–350)	Wills et al. (2002)
France	c.460dupA	p.Arg154LysfsX27	Dystonia, dysarthria, chorea, parkinsonism, blepharospasm, cerebellar signs	N.R.	Chinnery (2003)
North of UK	c.460dupA	p.Arg154LysfsX27	Extrapyramidal dysfunction	3–23 (25–400)	Crompton (2005)
South of UK	c.460dupA	p.Arg154LysfsX27	Generalized dystonia, psychiatric symptoms	30 (18–300)	Mir (2005)
France	c.497_498dup	p.Phe167SerfsX26	Tremor, cerebellar ataxia, parkinsonism and pyramidal signs, behavioral disturbances, cognitive dysfunction	Normal	Vidal (2003)
Gypsy ancestry	c.285G > A	p.Ala96Thr	Parkinsonism, ataxia, and corticospinal signs	16 (20–300)	Maciel (2005)
French Canadian and Dutch ancestry	c.442dupC	p.His148ProfsX33	Dystonia, dysarthria, chorea, blepharospasm, cerebellar signs and mitochondrial respiratory chain defects	14 (10–291)	Mancuso (2005)
Japan	c.469_484dup	p.Leu162ArgfsX24	Tremor, hypotonia, hyperextensibility, aphonia and cognitive impairment	5 (33–330)	Ohta et al. (2008)
France	c.458dupA	p.His153GlnfsX28	Dystonia, dysarthria, dysphagia	N.R.	Devos et al. (2009)
Japan	c.439_442dup	p.His148ArgfsX34	Chorea, tremor, dyskinesia, dysarthria, dysphagia	46 (40–200)	Kubota et al. (2009)
Italy	c.469_484dup	p.Leu162ArgfsX24	Axial ataxia, severe dysarthria, dystonia, bilateral hand tremor, parkinsonism	3 (17–400)	Storti et al. (2013)
Japan	c.468_483dup	p.Leu162TrpfsX24	Chronic headache, orolingual dystonia, disarthria, cerebellar ataxia, pyramidal tract signs, psychiatric symptoms	20 (5–204)	Nishida et al. (2014)
France	c.468dupT	p.Gly157TrpfsX24	Dystonia, dysarthria, dysphagia,dysmetria	49 (80–250)	Moutton et al. (2014)

DNA mutations (a) and protein variants (b) are named according to the HGVS nomenclature guidelines (den Dunnen 2000). Nucleotides and aminoacid numbers correspond to CCDS database entry 33070.1.

c: Serum ferritin values were reported in μg/L, in brackets the reference ranges for normal individuals,

N.R. = not reported, Normal = value in normal range.
